# Emergence of Oseltamivir-Resistant Pandemic (H1N1) 2009 Virus within 48 Hours

**DOI:** 10.3201/eid1610.100688

**Published:** 2010-10

**Authors:** Masafumi Inoue, Timothy Barkham, Yee-Sin Leo, Kwai-Peng Chan, Angela Chow, Christopher W. Wong, Raphael Tze-Chuen Lee, Sebastian Maurer-Stroh, Raymond Lin, Cui Lin

**Affiliations:** Author affiliations: Agency for Science, Research and Technology, Singapore (M. Inoue, C.W. Wong, R.T.-C. Lee, S. Maurer-Stroh);; Tan Tock Seng Hospital, Singapore (T. Barkham, A. Chow);; Communicable Disease Center, Singapore (Y.-S. Leo);; Singapore General Hospital, Singapore (K.-P. Chan);; Ministry of Health, Singapore (R. Lin, L. Cui)

**Keywords:** Influenza, pandemic (H1N1) 2009, viruses, oseltamivir, antimicrobial resistance, Singapore, drug resistance, dispatch

## Abstract

An oseltamivir-resistant influenza A pandemic (H1N1) 2009 virus evolved and emerged from zero to 52% of detectable virus within 48 hours of a patient’s exposure to oseltamivir. Phylogenetic analysis and data gathered by pyrosequencing and cloning directly on clinical samples suggest that the mutant emerged de novo.

Early descriptions of emergence of H275Y mutants in pandemic (H1N1) 2009 virus showed resistance after 11 and 23 days of therapy in immunosuppressed patients (*1*). Also in previous reports, transmission of mutant viruses occurred in immunosuppressed patients (*2*), although a cluster among healthy persons demonstrated that H275Y mutants could replicate and cause disease in the absence of drug pressure (*3*). Additional reports noted decreasing times to detection of resistance, from 14 to 4 days after therapy (*4**–**6*). We report development of oseltamivir-resistant pandemic (H1N1) 2009 virus in an infected woman in Singapore within 48 hours of drug treatment.

## The Study

Pandemic (H1N1) 2009 virus was first detected in Singapore in May 2009. Infected patients were placed in isolation and offered oseltamivir, and respiratory samples were collected for screening for H275Y, the principal mutation associated with oseltamivir resistance in influenza A N1 viruses. H275Y was detected in a pandemic (H1N1) 2009 virus isolated from a sample from a 28-year-old female patient on the sixth day of illness within 48 hours of her exposure to oseltamivir. (Written patient consent was obtained under Review Board approval no. E09-230.) A sore throat, myalgia, redness of the right eye, and a mild fever with a productive cough had developed on the day she returned to Singapore from Hawaii. Eleven close contacts, exposed before emergence of the mutant, were given oseltamivir prophylaxis on the patient’s fourth day of treatment, and they remained well. By performing sequencing directly on 6 of her respiratory samples and on their viral isolates (Technical Appendix), we investigated the origin of this H275Y mutant (the second earliest sample of this mutation to be deposited in GenBank).

Only wild-type sequences were detected in samples collected on the day before, the day of, and 14 hours after initiation of oseltamivir therapy (Technical Appendix Table 1). Similarly, only wild-type sequences were detected in 192 clones generated from a sample collected a few hours before initiation of oseltamivir. Pyrosequencing directly on clinical samples collected 38 and 45 hours after initiation of therapy showed 24% and 52% mutant sequences, respectively (Figure). The relative amount of virus detected, as determined by the strength of PCR results (Technical Appendix Table 1), increased from days 3 to 5 of illness by ≈1,000-fold. Oseltamivir treatment was initiated on day 4 of illness. On the same day, her maximum body temperature (38.8°C) was recorded, although no other signs or symptoms of clinical deterioration were observed. Her fever resolved on day 5 of illness, and she was allowed out of isolation on day 7 of illness.

**Figure:**
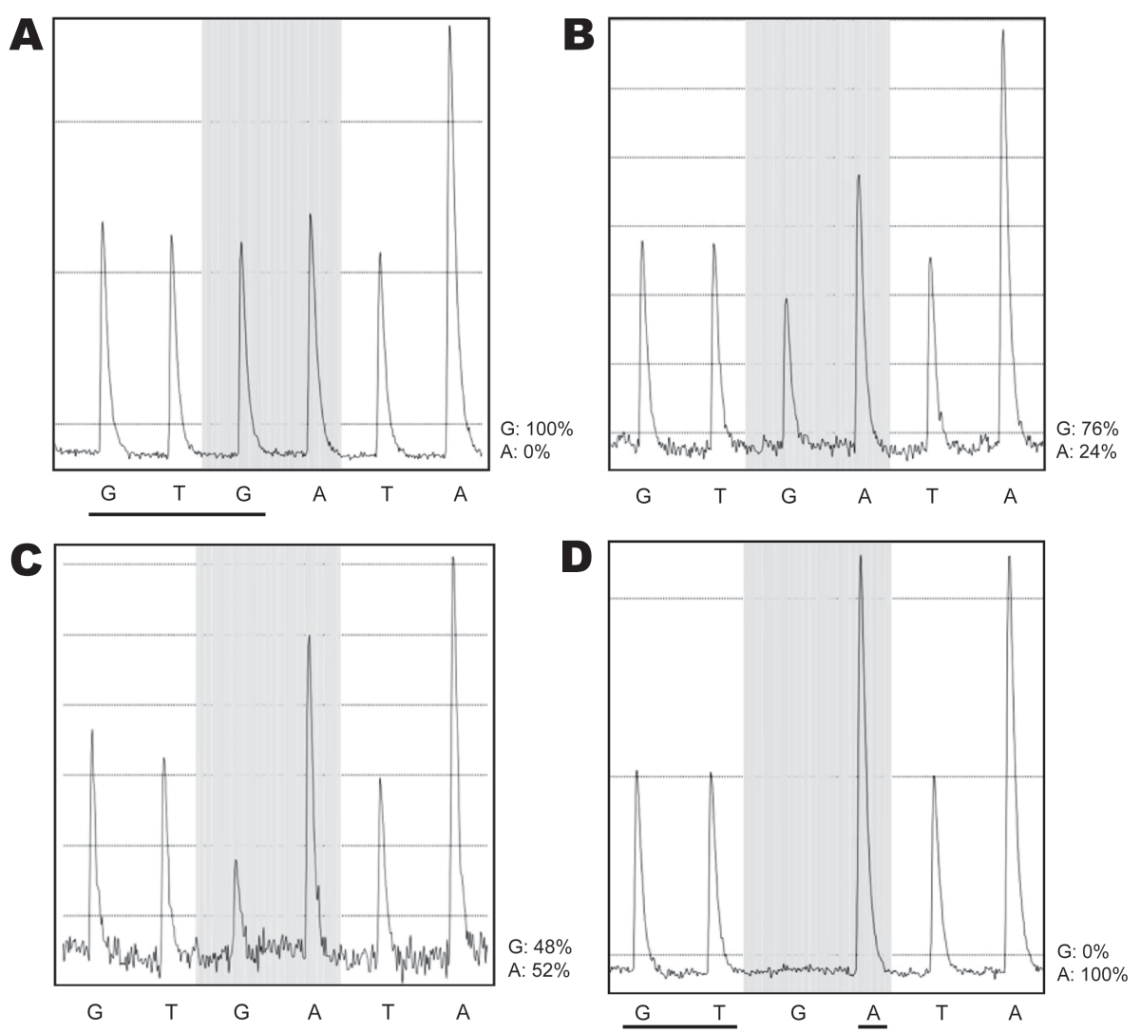
Pyrograms showing evolution of the H275Y mutation in pandemic (H1N1) 2009 virus, Singapore. A) May 29, sample 14 h after receiving oseltamivir shows 100% G. B) May 30, 38-h sample shows 76% G and 24% A. C) May 30, sample at 45 h shows 48% G and 52% A. D) May 30, virus isolated from 38-h sample is 100% A. The shaded area indicates the mutation site, showing the progressive loss of the third base, G, and its replacement by A. In panel A, all bases have peaks of equivalent height because each base is a singlet, except for the last peak, which is double the height and represents an A followed by another A, as in AA. In panel D, the G at the mutation site has disappeared and the signal of the next base, A, has doubled in amplitude, indicating the complete replacement of G by A. This reflects replacement of the complementary base C by T in the viral template. The 3 bases that constitute aa 275 in the neuraminidase protein are underlined in panels A and D. In panel A, the sequence is 100% GTGATAA. In panel D, it is 100% GTAATAA. In panel A, the wild-type 5′-GTG-3′ is equivalent to 3′-CAC-5′ in its plus-strand RNA, which codes for histidine (H). Similarly, the mutant 5′-GTA-3′ in panel D is equivalent to 3′-CAT-5′, which codes for tyrosine (Y). Therefore, the pyrograms show a mutation from H to Y at position 275—the H275Y mutant.

When we compared the mutant drug-resistant isolate GN285 with the wild-type drug-sensitive isolate ON129, we found only 1 aa difference, the H275Y resistance-causing mutation in the neuraminidase gene, whereas a comparison of GN285 and ON129 with the reference strain A/Texas/05/2009(H1N1) showed several mutations (Technical Appendix Table 2). Mutation PB1 I435V, shared between GN285 and ON129, did not occur in any of the other 7 drug-resistant strains included in the analysis. The whole genome maximum likelihood tree (Technical Appendix Figure 1 ) showed that the wild-type and resistant viruses isolated from this patient were more closely related to each other than to any other virus in the analysis. Notably, GN285 and ON129 clustered together in 376 of the 500 bootstrap tests.

## Conclusions

Our data indicate that oseltamivir resistance developed within 2 days. This time is similar to the interval for development of resistance to adamantanes in subtype H3N2 viruses when 30% of treated patients shed resistant, transmissible virus within 3 days of beginning treatment (*7*). Four cases of H275Y infection have been detected, with the use of sequencing, among 1,060 pandemic (H1N1) 2009 isolates tested (0.47%) in Singapore since June 2009 (not including the case reported here). Only 1 case was found in a pretreatment sample; the other 3 were identified after treatment. The emergence of H275Y might also be affected by the timing of therapy. In the case reported here, oseltamivir treatment was begun on day 4 of illness when viral titers were almost maximal, which is probably the stage of illness best suited to select for resistant mutants because the presence of mutations is likely to be greatest when replication is greatest. Notably, rates of H275Y are high, reaching 13% (*8*) among immunocompromised groups in whom high viral titers might also be a contributory factor.

Pyrosequencing directly on clinical material allows the measurement of relative quantities of viral variants without introducing errors inherent in viral culture, which is known to favor the growth of H275Y mutants (*6*). Pyrosequencing cannot exclude the presence of subpopulations of <5%–10%, but if small proportions of mutant virus had been present, they would have been detected in the culture of the sample collected 14 hours after exposure to oseltamivir; however, only wild-type sequences were detected. Similarly, only wild-type sequences were detected in 192 clones derived directly from the sample collected a few hours before the first dose of oseltamivir. The phylogenetic data show, at the amino acid and nucleotide level, that the resistant and sensitive isolates cluster together, apart from other viruses. These data support the hypothesis that the H275Y mutant arose de novo from the wild-type virus from the same patient.

The fact that the effects of oseltamivir are likely to be greatest in severe disease (*9**–**11*), but of modest benefit in mild infections (*12*), has led to proposals for restricting the use of antiviral agents and the use of alternative antiviral agents and multidrug therapy to prevent the emergence of resistance (*7**,**9*) in severe cases. The proposed interventions may be of little consequence compared with the association and co-selection of H275Y with other genetic determinants. Although in isolation H275Y compromises seasonal influenza (H1N1) by reducing the amount of neuraminidase expressed on the cell surface, other mutations (R194G, V234M, and R222Q) may compensate and restore its expression to levels found in wild-type virus, without H275Y (*13*). This circumstance may explain the emergence and spread of H275Y in the absence of drug pressure in seasonal influenza (H1N1), which increased from being negligible in 2007 to 95% in March 2009 (*7*) despite a low consumption of oseltamivir (*14*). More than 99% of all pandemic (H1N1) 2009 neuraminidases have G at position 194, which corresponds to the R194G in seasonal influenza (H1N1). However, the effects of mutations are not easily transferable among different influenza (H1N1) types and may need to be tested separately (Technical Appendix ).

The clinical effects of the spread of H275Y in seasonal influenza (H1N1) have been minimal because seasonal influenza (H1N1) now accounts for an insignificant proportion of influenza (*15*). However, if H275Y mutants of pandemic (H1N1) 2009 emulate the expansion of resistant seasonal influenza (H1N1), the effect might be substantial because pandemic (H1N1) 2009 accounted for 90%–95% of circulating influenza A viruses in the Northern and Southern Hemispheres in late 2009 (*15*). We are speculating, however, because our laboratory data show that pandemic (H1N1) 2009 fell from 62% to 29% of 436 influenza cases from May 2010 to mid June 2010, Singapore’s main influenza season, whereas the presence of influenza (H3N2) has risen from 23% to 53% (influenza B accounts for 15%–20%). As the next influenza season in the Southern Hemisphere approaches, the relative mixture of subtype H3N2 and H1N1 viruses will be under scrutiny again, not only to predict “best bet” vaccine components but also to ascertain their associated resistance patterns. Whatever the epidemiologic data exhibit, clinicians should consider resistance when patients do not respond to treatment for pandemic (H1N1) 2009 because H275Y can emerge literally overnight, as the case reported here reminds us.

## Supplementary Material

Technical Appendix Experimental Methods, Results for 6 respiratory samples collected, Amino acid differences (mutations), Maximum-likelihood phylogenetic tree, and the Effects of Permissive Mutations.

## References

[1] Centers for Disease Control and Prevention. Oseltamivir-resistant novel influenza A (H1N1) virus infection in two immunosuppressed patients—Seattle, Washington, 2009. MMWR Morb Mortal Wkly Rep. 2009;58:893–6. 19696719

[2] Oseltamivir resistance in immunocompromised hospital patients. World Health Organization pandemic (H1N1) 2009 briefing note 18, 2009 Dec 2 [cited 2010 Apr 29]. http://www.who.int/csr/disease/swineflu/notes/briefing_20091202/en/index.html

[3] Le QM, Wertheim HF, Duong TN, van Doorn HR, Tran Hien N, Horby P. the Vietnam H1N1 Investigation Team. A community cluster of oseltamivir-resistant cases of 2009 H1N1 influenza. N Engl J Med. 2010;362:86–7. 10.1056/NEJMc0910448 20007549

[4] Memoli MJ, Hrabal RJ, Hassantoufighi A, Eichelberger MC, Taubenberger JK. Rapid selection of oseltamivir- and peramivir-resistant pandemic H1N1 virus during therapy in 2 immunocompromised hosts. Clin Infect Dis. 2010;50:1252–5. 10.1086/651605 20345239 PMC2946636

[5] Esposito S, Molteni CG, Colombo C, Daleno C, Daccò V, Lackenby A, Oseltamivir-induced resistant pandemic A/H1N1 influenza virus in a child with cystic fibrosis and *Pseudomonas aeruginosa* infection. J Clin Virol. 2010;48:62–5. 10.1016/j.jcv.2010.02.019 20335065

[6] Sy CL, Lee SSJ, Liu MT, Tsai HC, Chen YS. Rapid emergence of oseltamivir resistance. Emerg Infect Dis. 2010;16:723–5. 20350402 10.3201/eid1604.091706 PMC3321971

[7] Moscana A. Global transmission of oseltamivir-resistant influenza. N Engl J Med. 2009;360:953–6. 10.1056/NEJMp0900648 19258250

[8] Tramontana AR, George B, Hurt AC, Doyle JS, Langan K, Reid AB, Oseltamivir resistance in adult oncology and hematology patients infected with pandemic (H1N1) 2009 virus, Australia. Emerg Infect Dis. 2010;16:1068–75. 10.3201/eid1607.091691 20587176 PMC3321901

[9] Poland GA, Jacobson RM, Ovsyannikova IG. Influenza virus resistance to antiviral agents: a plea for rational use. Clin Infect Dis. 2009;48:1254–6. 10.1086/598989 19323631 PMC2831648

[10] McGeer A, Green KA, Plevneshi A, Shigayeva A, Siddiqi N, Raboud J, ; Toronto Invasive Bacterial Diseases Network. Antiviral therapy and outcomes of influenza requiring hospitalization in Ontario, Canada. Clin Infect Dis. 2007;45:1568–75. 10.1086/523584 18190317

[11] Dominguez-Cherit G, Lapinsky SE, Macias AE, Pinto R, Espinosa-Perez L, de la Torre A, Critically ill patients with 2009 influenza A (H1N1) in Mexico. JAMA. 2009;302:1880–7. 10.1001/jama.2009.1536 19822626

[12] Jefferson T, Jones M, Doshi P, Del Mar C. Neuraminidase inhibitors for preventing and treating influenza in healthy adults: systematic review and metaanalysis. BMJ. 2009;339:b5106. 10.1136/bmj.b5106 19995812 PMC2790574

[13] Bloom JD, Gong LI, Baltimore D. Permissive secondary mutations enable the evolution of influenza oseltamivir resistance. Science. 2010;328:1272–5. 10.1126/science.1187816 20522774 PMC2913718

[14] Kramarz P, Monnet D, Nicoll A, Yilmaz C, Ciancio B. Use of oseltamivir in 12 European countries between 2002 and 2007—lack of association with the appearance of oseltamivir-resistant influenza A (H1N1) viruses. Euro Surveill. 2009;14:1–5. 10.2807/ese.14.05.19112-en 19215715

[15] FluNet, Global Influenza Surveillance Network (GISN). Global circulation of influenza viruses. Number of specimens positive for influenza by subtypes from week no. 17 (2009) to 21 (2010) [cited 2010 Jun 16]. http://www.who.int/csr/disease/swineflu/Virologicaldata2010_06_11.pdf

