# Supplementary material for: Emergence of Oseltamivir-Resistant Pandemic (H1N1) 2009 Virus within 48 Hours
Source: Emerg Infect Dis. 2010 Oct;16(10):1633–6. doi: 10.3201/eid1610.100688 (PMC3294403; doi:10.3201/eid1610.100688)
Supplement: Technical Appendix — Experimental Methods, Results for 6 respiratory samples collected, Amino acid differences (mutations), Maximum-likelihood phylogenetic tree, and the Effects of Permissive Mutations. [file 10-0688-Techapp_6p.pdf]

Article DOI: 10.3201/eid1610.100688

Emergence of Oseltamivir-Resistant Pandemic (H1N1) 2009 Virus Within 48 Hours

Technical Appendix

Experimental Methods

Clinical data and daily combined nasal and throat swab specimens were collected prospectively in universal transport medium. Nucleic acids were extracted by using easyMag (bioMérieux, Marcy-l’Etoile, France); aliquots were frozen at –80°C. Real- time reverse transcription–PCR (RT-PCR) for influenza A (H1N1) 2009 was carried out with an in-house assay on an Mx3005P instrument (Stratagene, La Jolla, CA, USA). Virus isolation was carried out with Madin-Darby canine kidney cells. RNA extracted directly from samples and from culture supernatants was sequenced. The full genome of the wild-type isolate, A/Singapore/ON129/2009(H1N1), was sequenced, according to published methods (*1*) and, among the 8 segments, the nucleoprotein and RNA polymerase B segments were resequenced with a protocol from the World Health Organization Collaborating Center for Influenza at the Centers for Disease Control and Prevention (Atlanta, GA, USA) (http://www.who.int/csr/resources/publications/swineflu/pyrosequencing_protocol/en/index.html). Their GenBank accession nos. are CY049065, CY049066, CY049067, CY049068, CY049069, CY049070, CY049071, and CY049072. The resistant isolate /Singapore/GN285/2009(H1N1) was sequenced with the same Centers for Disease Control and Prevention protocol. The GenBank accession nos. are CY055300, CY055301, CY055302, CY055303, CY055304, CY055305, CY055306, and CY055307. A PCR fragment ≈1 kb (obtained directly, without culture, from the clinical sample collected a few hours before initiation of oseltamivir on day 4 of illness) containing the aa position 275 on neuraminidase (NA) (the mutation point) was cloned into a pGEM-T vector (Promega Corp., Madison, WI, USA), and 192 clones picked and sequenced.

Creation of Amplicons for Pyrosequencing

RT-PCR was performed with the SuperScript III One-Step RT-PCR System with Platinum Taq (cat. no: 12574-018; Invitrogen Life Technologies, Carlsbad, CA, USA) in a 50-µL reaction volume containing 5 µL of RNA sample. The biotinylated PCR product was generated with forward primer, (5'-Bio/TGCTTTACTGTAATGACCGAT-3') at a final concentration of 0.25 µmol/L and reverse primer, (5'-GATTCTGGTTGAAAGACACCC-3') at a final concentration of 0.2 µmol/L in a thermal cycler. The resulting 217-bp amplicon accommodates the codon for H275 (CAC) or H275Y (TAC) in the product. A Mastercycler-ep-gradient-S (Eppendorf, Hamburg, Germany) was used with the following steps and conditions: reverse transcription at 55°C for 10 min and initial denaturation at 94°C for 2.5 min; followed by 40 cycles of denaturation at 94°C for 32 s, annealing at 57°C for 76 s, and extension at 68°C for 33 s; and a final extension at 68°C for 5 min. PCR products were analyzed by gel electrophoresis to estimate the yield.

Pyrosequencing

Pyrosequencing was performed according to the manufacturer's guidelines (Biotage, Uppsala, Sweden). Briefly, ≈200 ng of biotinylated PCR product was reacted with streptavidin-coated beads (GE Healthcare, Little Chalfont, UK) by shaking at room temperature for 15 min, followed by collecting DNA-coated beads by vacuum onto a 96-well vacuum tool and serially immersing the beads into 70% ethanol, 0·2 mol/L NaOH, and 10 mmol/L Tris-acetate, pH 7.6, washing buffer. The beads with single-stranded DNA template were released into each well of PSQ96-well plate (Biotage) with 40 µL of annealing buffer (20 mmol/L Tris-acetate, pH 7·6, and 2 mmol/L magnesium acetate) containing the sequencing primer (5'-TAGAATCAGGATAACAGGAGCA-3') at a final concentration of 0.4 µmol/L. The plate was heated at 80ºC for 2 min and then cooled to room temperature for 10 min before being placed into the PyroMark Q96 ID System (Biotage). The sequencing procedure was performed at room temperature by cyclic dispensation of substrates, enzymes, and 4 dNTPs (Biotage) in a prespecified order to enable single nucleotide polymorphism analysis and generation of quantitative data. We sequenced a 25-bp region that included the H275Y mutation of NA. Relative proportions of bases, expressed as a percentage, were determined by using the PyroMark instrument.

The description of the mutation as H275Y means that the mutation in the gene results in the original amino acid at position 275 along NA (in N1 numbering) changing from the expected wild-type histidine (H) to tyrosine (Y). The actual change in the NA gene is a single point mutation from a cytosine base to a thymidine base.

Phylogenetic Analysis

All 7 strains with the NA H275Y mutation for which the whole genome (10 genes from 8 segments) was available from the National Center for Biotechnology Information (Bethesda, MD, USA) influenza virus resource (*2*) were compared. A drug-sensitive isolate (ON141), which was geographically and temporally closely related (a difference of <1 week) with full genome available, was also included. Because the nucleotide level also enables seeing synonymous nucleotide exchanges (without an amino acid change) that could harbor additional similarity information, we chose to derive a maximum likelihood tree over the whole coding genome to investigate the relationship of these 10 strains in further detail. Nucleotide alignments of the coding regions of all 10 genes in the 8 segments of the 10 strains under study were concatenated. A maximum likelihood tree was calculated with PhyML3 (*3*) by using the following parameters: tree search = NNIs; initial tree = BIONJ; model of nucleotides substitution = HKY85; log-likelihood = -18939.44126; discrete gamma model = yes, number of categories = 4, gamma shape parameter = 97.954 (estimated by PhyML); proportion of invariant = 0.923, transition/transversion ratio = 16.943; bootstrap steps = 500. The final tree was displayed and exported from MEGA4 (*4*).

Technical Appendix Table 1. Results for 6 respiratory samples collected from patient infected with pandemic (H1N1) 2009 over 4 days, Singapore*

Characteristic						
Day of illness	3	3	4	5	6	6
Sample date in May 2009	27	27	28	29	30	30
Hours after oseltamivir initiated	–	–	0	14	38	45
Oseltamivir, 75 mg	None	None	1 dose	2 doses	2 doses	2 doses
RT PCR, C_t value	32	33	29	24	32	35
Pyrosequencing directly on sample, %	100	100	100	100	76	48
Wild type						
Mutant	0	0	0	0	24	52
Sequencing of 192 cloned fragments obtained directly from the clinical sample, %	–	–	100	–	–	–
Wild-type						
Mutant	–	–	0	–	–	–
BigDye sequencing directly on clinical sample, %	–	–	100	–	–	–
Wild type						
Mutant	–	–	0%	–	–	–
Viral isolation	–	–	Positive	Positive	Positive	–
BigDye sequencing of isolated virus, %	–	–	100	100	0	–
Wild-type						
Mutant	–	–	0	0	100	–
Pyrosequence of isolated virus, %	–	–	100	100	0	–
Wild type						
Mutant	–	–	0	0	100	–

*RT-PCR, reverse transcription PCR for Influenza A (H1N1) 2009; C_t, cycle threshold; mutant, H275Y mutation –, not done/not applicable; .

Technical Appendix Table 2. Amino acid differences (mutations) between the 10 viral isolates included in the phylogenetic analysis compared with the early reference strain A/Texas/05/2009(H1N1)*

Strain	Protein									
	PB2	PB1	PA	HA	NP	NA	M1	M2	NS1	NS2
A/Iwate/3/2009 (H1N1)				S220T	V100I	V106I, N248D, H275Y	K113R		I123V	
A/Yamaguchi/22/2009 (H1N1)			V379I	S220T	V100I	V106I, N248D, H275Y			I123V	
A/Hunan/SWL3/2009 (H1N1)	S155N, M243L, I398V		T357A		K90R, V100I	V106I, N248D, H275Y			N48K, V60I, D101E	
A/Quebec/147365/2009 (H1N1)			A20T, L686V	S220T, A273S	V100I	V106I, N248D, H275Y			M79T, I123V	
A/Denmark/528/2009 (H1N1)		F730S		S220T	V100I	V106I, N248D, H275Y			M93I, I123V	
A/Tokushima/2/2009 (H1N1)			M311I	S220T	V100I	V106I, N248D, H275Y			I123V	
A/Osaka/180/2009 (H1N1)	V649I, E700K	I667T		V169I	V100I	V106I, H275Y	A33T			E63K
A/Singapore/GN285/2009 (H1N1)		I435V		S220T	V100I	V106I, N248D, H275Y			I123V	
A/Singapore/ON129/2009 (H1N1)		I435V		S220T	V100I	V106I, N248D			I123V	
A/Singapore/ON141/2009 (H1N1)	K482R			S220T, R238G	V100I	A86V, V106I, N248D			I123V	

* PB1, PB2, polymerase basic 1 and 2; PA, polymerase acidic; HA, hemagglutinin; NP, nucleoprotein; NA, neuraminidase; M1, M2, matrix 1 and 2; NS1, NS2, nonstructural 1 and 2.

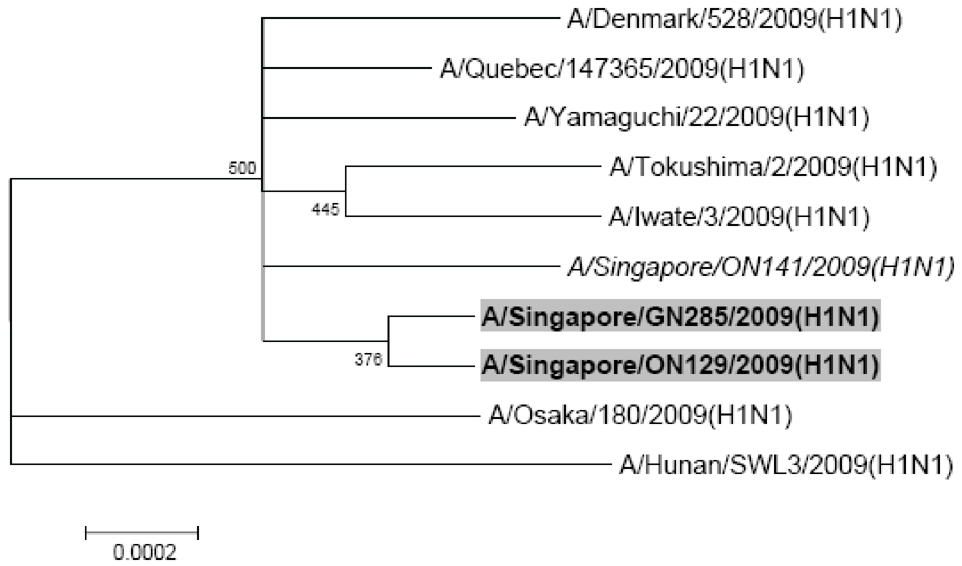

Technical Appendix Figure. Maximum-likelihood phylogenetic tree. Shaded strains are oseltamivir susceptible (Singapore/ON129) and resistant (Singapore/GN285) isolates from the case presented. Singapore/ON141 is a drug-sensitive isolate geographically and temporally closely related to ON129 and GN285 is shown in italics. The remaining strains in the tree are all isolates with the H275Y mutation. Scale bar indicates nucleotide substitutions per site.

Effects of Permissive Mutations

A/Singapore/GN285/2009(H1N1) and 99.9% of all pandemic (H1N1) 2009 NAs have G at position 194, which would correspond to the R194G mutation that resulted in rescue of the expression deficit caused by the H274/275Y mutation in the context of the WSN laboratory strain. However, as shown in Figure 2B in the article by Bloom et al. (*5*), there remains an expression deficit with the H274/275Y mutation in the context of A/California/04/2009(H1N1), which also has G at position 194. Consequently, the results and effects of the respective mutations are not easily transferable among different H1N1 subtypes and may need to be tested separately.

For completeness, the most typical amino acid at position 222 among the pandemic NAs is N, which is physicochemically similar to the Q of the R222Q mutation. However, the 2 preceding residues also differ between seasonal and pandemic NAs, which may alter the local structure and consequently potential effects on other positions. At position 234, most pandemic

(H1N1) 2009 sequences (including those of A/Singapore/GN285/2009[H1N1] and A/California/04/2009[H1N1]) have the nonpermissive V. Effects of the NA mutations V106I and N248D on expression of the virus are not known at present, but because of their common occurrence in later phases of the pandemic (thus far), they should not be considered unfavorable for the virus.

References

1. Lee WH, Koh CW, Chan YS, Aw PK, Loh KH, Han BL, et al. Large-scale evolutionary surveillance of the 2009 H1N1 influenza A virus using resequencing arrays. Nucleic Acids Res. 2010;38:e111. . PubMed DOI: 10.1093/nar/gkq089

2. Bao Y, Bolotov P, Dernovoy D, Kiryutin B, Zaslavsky L, Tatusova T, et al. The influenza virus resource at the National Center for Biotechnology Information. J Virol. 2008;82:596–601.

3. Guindon S, Gascuel O. A simple, fast, and accurate algorithm to estimate large phylogenies by maximum likelihood. Syst Biol. 2003;52:696–704. PubMed DOI: 10.1080/10635150390235520

4. Tamura K, Dudley J, Nei M, Kumar S. MEGA4: Molecular Evolutionary Genetics Analysis (MEGA) software version 4.0. Mol Biol Evol. 2007;24:1596–9. PubMed DOI: 10.1093/molbev/msm092

5. Bloom JD, Gong LI, Baltimore D. Permissive secondary mutations enable the evolution of influenza oseltamivir resistance. Science. 2010 328:1272–5.
